# Movement disorders in hereditary spastic paraplegia (HSP): a systematic review and individual participant data meta-analysis

**DOI:** 10.1007/s10072-022-06516-8

**Published:** 2022-11-28

**Authors:** Seyed-Mohammad Fereshtehnejad, Philip A. Saleh, Lais M. Oliveira, Neha Patel, Suvorit Bhowmick, Gerard Saranza, Lorraine V. Kalia

**Affiliations:** 1 Division of Neurology, Department of Medicine, University of Ottawa, Ottawa, ON Canada; 2 Division of Clinical Geriatrics, Department of Neurobiology, Care Sciences and Society (NVS), Karolinska Institutet, Stockholm, Sweden; 3 Edmond J. Safra Program in Parkinson’s Disease and Morton and Gloria Shulman Movement Disorders Clinic, Toronto Western Hospital, UHN, Toronto, ON Canada; 4 Division of Neurology, Department of Medicine, University of Toronto, Toronto, ON Canada; 5 Krembil Research Institute, Toronto Western Hospital, University Health Network, Toronto, ON Canada

**Keywords:** Hereditary spastic paraplegia (HSP), Movement disorders, Genotype, Phenotype, *SPG* (SPastic parapleGia)

## Abstract

**Background:**

Hereditary spastic paraplegia (HSP) is a rare genetic disorder associated with mutations in > 80 loci designated *SPG* (SPastic parapleGia). The phenotypic spectrum of HSP can extend to include other neurologic features, including movement disorders. Our aim was to investigate genotype–phenotype associations in HSP with a focus on movement disorders.

**Methods:**

We performed a systematic review and individual participant data (IPD)-level meta-analysis by retrieving publications from Medline/EMBASE/Web of Science on HSP with a SPG genotype. Studies were included only if individual-level information was accessible and at least one patient with a movement disorder was reported for that genotype. Out of 21,957 hits, 192 manuscripts with a total of 1413 HSP cases were eligible. Data were compared between two HSP groups: manifested with (HSP-MD, *n* = 767) or without (HSP-nMD, *n* = 646) a movement disorder.

**Results:**

The HSP-MD group had an older age of onset (20.5 ± 16.0 vs. 17.1 ± 14.2 yr, *p* < 0.001) and less frequent autosomal dominant inheritance (7.6% vs. 30.1%, *p* < 0.001) compared to HSP-nMD. SPG7 (31.2%) and SPG11 (23.8%) were the most frequent genotypes in the HSP-MD group. HSP-MD with SPG7 had higher frequency of later onset during adulthood (82.9% vs. 8.5%), ataxia (OR = 12.6), extraocular movement disturbances (OR = 3.4) and seizure (OR = 3.7) compared to HSP-MD with SPG11. Conversely, SPG11 mutations were more frequently associated with consanguinity (OR = 4.1), parkinsonism (OR = 7.8), dystonia (OR = 5.4), peripheral neuropathy (OR = 26.9), and cognitive dysfunction (OR = 34.5).

**Conclusion:**

This systematic IPD-level meta-analysis provides the largest data on genotype–phenotype associations in HSP-MD. Several clinically relevant phenotypic differences were found between various genotypes, which can possibly facilitate diagnosis in resource-limited settings.

**Supplementary Information:**

The online version contains supplementary material available at 10.1007/s10072-022-06516-8.

## Introduction

Hereditary spastic paraplegia (HSP) is a large and diverse group of genetic disorders primarily associated with corticospinal tract dysfunction. HSP is highly heterogeneous—both clinically and genetically—and contributes to challenging landscapes in clinical practice. It has been traditionally classified as pure/uncomplicated, when pyramidal tract signs are prominent and sometimes accompanied by sensory or urinary symptoms, or complex/complicated, when additional features such as seizures and cognitive impairment are present [1]. Distinct modes of inheritance have been described (autosomal dominant, autosomal recessive, X-linked, and rarely, mitochondrial), and over 80 distinct genetic loci have been linked to HSP [2, 3]. Mutations in the *SPAST* (*SPG4*) gene are the most frequent cause of autosomal dominant HSP, while *SPG11* mutations are responsible for most autosomal recessive forms [2].

Movement disorders, such as ataxia, parkinsonism, and dystonia, can be accompanying neurological features in HSP. In fact, genes responsible for hereditary ataxias overlap with HSP genes, and ataxia-spasticity may be considered along a continuous disease spectrum [4, 5]. For instance, ataxia is a major clinical presentation in patients with *SPG7* mutations [6, 7]. Parkinsonism—sometimes l-dopa responsive—and dystonia have been described in *SPG11* [2, 8–11] and *SPG15* [12, 13], while cervical dystonia, spasmodic dysphonia, and limb dystonia have been reported in *SPG7* [14–16]. Myoclonus is a rare feature of HSP [17]. While these previous studies have started to delineate associations between specific SPG loci and movement disorders, a large but focused effort is needed to draw stronger, more accurate, and potentially predictive correlations.

Our aim is to systematically review and perform an individual participant data-level meta-analysis on HSP with known *SPG* genotype, exploring the prevalence and type of movement disorders and other clinically relevant phenotypic differences in patients with distinct genotypes. In view of the wide HSP phenotypic heterogeneity, we suspect the presence and type of movement disorder may aid in the diagnosis of this complex disorder.

## Methods

### Data sources and searching strategy

**Fig. 1:**
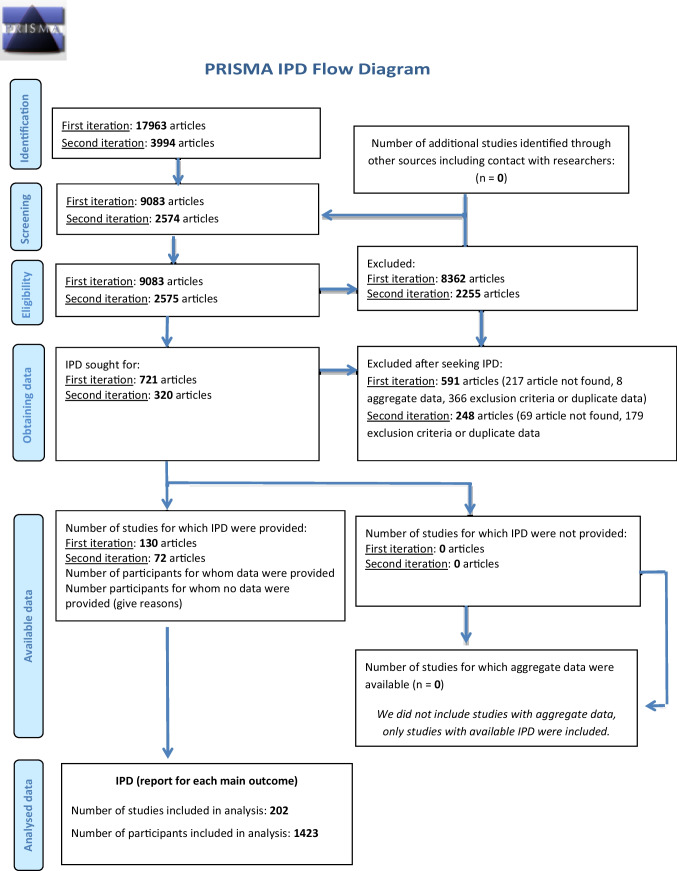
The PRISMA-IPD flow diagram

We performed a systematic review of available literature, according to the Preferred Reporting Items for Systematic Review and Meta-Analyses of Individual Participant Data (the PRISMA-IPD) guideline [18], on HSP with a known SPG genotype published from 1990 to June 2018 (flow diagram in Fig. 1). A research librarian conducted literature searches of three major scientific databases (Medline, EMBASE, and Web of Science (SCI-EXPANDED, SSCI, A&HCI, CPCI-S, CPCI-SSH, ESCI) using relevant database-specific subject headings and keywords (Supplementary Material). The initial search was performed in September 2015 and an update search was run June 2018. All articles published prior to 1990 were excluded. Duplicate articles were removed. Abstract screening of the remaining articles was performed by three separate investigators (S. M. F., P. A. S., N. P.). Lists of included articles were then compared, and a final decision as to include or exclude a particular article was made by the three investigators (S. M. F., P. A. S., N. P.). In case of discrepancy between the investigators, a fourth investigator (L. V. K.) decided.

### Abstract screening

Abstracts were included if reference was made to a clinical description of at least one HSP patient with a numerically defined loci with an SPG designation according to McKusick’s Online Mendelian Inheritance in Man (OMIM®) [19]. Mitochondrial HSP is extremely rare, and we did not encounter any SPG designation associated with mutations in the mitochondrial genome. Articles were excluded if they were published prior to 1990. Review articles and non-human studies were also excluded. For all included abstracts and all potentially relevant articles without an abstract, we attempted to access full-text articles through the University of Toronto library. Articles were excluded if not found or if published in a language other than English and an English translation was unavailable.

### Full-text screening

Among the full-text articles, case reports, case series, cross-sectional studies, and retrospective chart review articles were all eligible for inclusion. Eligible articles were read and included only if individual-level information was accessible for every patient and at least one patient with a movement disorder was reported. Movement disorders were defined as ataxia, dystonia, parkinsonism, rest tremor, action tremor (e.g., intention tremor, terminal tremor), myoclonus, chorea, or dyskinesia related or unrelated to dopaminergic therapy. Eligible articles also required the genetic confirmation of a suspected pathogenic variant of a *SPG* gene. In vitro studies and in silico studies were also excluded. Patients without pyramidal signs were excluded unless they had a movement disorder (e.g., ataxia). Patients with a known pathogenic *SPG* mutation but with a well-characterized non-HSP presentation (i.e., Pelizaeus Merzbacher disease, Gordon Holmes syndrome, and Boucher-Neuhauser syndrome) were also excluded. To avoid duplicating a case that was reported in more than one publication, articles referencing previously published cases were excluded and, during data extraction, we audited the datasheet by the names of the first authors and patients’ characteristics to identify and delete duplicates.

### Data extraction

Five investigators (S. M. F., P. A. S., N. P., S. B., G. S.) extracted manuscript characteristics (e.g., first authors name) and IPD for all reported cases from eligible articles that met the inclusion criteria. HSP cases within families were treated as individual cases and considered separately when meeting inclusion criteria and compiling data. Therefore, we included any other family member for whom the genotype (SPG designation) and individualized phenotypic information (clinical features) were reported. For articles with supplementary data, additional clinical and genetic data were extracted from the supplementary materials published with the articles. The IPD variables consisted of *SPG* gene, sex, age at symptom(s) onset, age at reported assessment, family history of HSP, initial presentation, motor features (i.e., pyramidal signs, motor weakness, gait or posture abnormalities, ataxia, dystonia, action tremor, rest tremor, parkinsonism, levodopa-responsiveness, motor fluctuations, dyskinesia, chorea, myoclonus), and non-motor features (i.e., cognitive impairment, speech disorder, peripheral neuropathy, urinary symptoms, seizure, extraocular movement abnormalities, retina/optic nerve abnormalities, dysphagia, deafness, sleep disorder, depression, skin lesions). We did not aim to include aggregate group-level data, and we did not contact authors of these manuscripts for IPD information.

### Data synthesis and analysis

Extracted IPD was pooled for statistical analysis. We divided the study population into two groups: (1) HSP patients with movement disorder (HSP-MD) and (2) HSP patients without movement disorders (HSP-nMD). Demographic, genotype (i.e., *SPG* mutation), and clinical data were then compared between the two groups. We used frequency percentages and mean (standard deviation (SD)) for description of categorical/nominal and numeric variables, respectively. Descriptive statistics were reported only for *SPG* subtypes with at least five reported cases. Differences in demographics, genotypes, and clinical characteristics between HSP-MD and HSP-nMD groups were statistically compared using chi-square or independent-samples *t* test where appropriate. For the HSP-MD group, Kendall’s correlation was used to explore pair-wise associations between various clinical presentations, and a corresponding correlation coefficient was calculated and reported as a percentage in the correlation matrix. We used the chi-square test to compare clinical features between the two most common *SPG* subtypes within the HSP-MD group (*SPG7* versus *SPG11*). For each significant difference, corresponding odds ratio (OR) and its 95% confidence interval (CI) were calculated. Logistic regression models were applied to adjust these between-group differences (*SPG7* versus *SPG11*) for disease duration as a covariate in each model, and to calculate adjusted ORs for each clinical feature. All analyses were performed using *R* (R, version 3.3.3; the R Core Development team, 2010) and *IBM SPSS* Statistics software (version 23.0). For all analyses, two-sided *p*-values < 0.05 were considered statistically significant.

## Results

IPD was extracted for 1423 patients with genetically confirmed HSP: 767 with movement disorders (HSP-MD) and 646 without movement disorders (HSP-nMND). The demographic features for all included patients are summarized in Table 1. HSP-MD and HSP-nMD patients were similar with respect to sex and family history of HSP. HSP-MD patients were more likely to have a later age of onset and thus an older age of assessment. Disease duration, defined as age at the time of clinical assessment minus age at symptoms onset, was slightly longer in HSP-MD group (18.3 (SD = 12.8) years vs. 16.1 (SD = 12.7) years, *p* = 0.004). Autosomal dominant inheritance was less common in the HSP-MD group. Gait abnormalities and spasticity were common initial presentations, although the former was more frequent among HSP-MD, and the latter was more frequent among HSP-nMD. Ataxia and cognitive impairment were also common initial presentations among HSP-MD and HSP-nMD, respectively.

**Table 1:** Demographic and baseline characteristics of recruited individuals with hereditary spastic paraplegia (HSP) manifested with (HSP-MD, *n* = 767) or without (HSP-nMD, *n* = 646) a movement disorder

Genotype	Sex Male (%)	Age of Onset (yr) Mean ± SD	Age at Visit (yr) Mean ± SD	Family history (%)	Inheritance (%)	Initial presentation* (%)
**HSP-MD** *(n* = *767)*	53.4%	20.5 ± 16.0 (range: < 1 mo–65 yr)	38.0 ± 17.9 (range: 1 mo–90 yr)	78.9%	AR (58.4%) AD (7.6%) AR/AD (31.4%) X-linked (2.6%)	Gait disturbance (50.0%) Ataxia (8.7%) Spasticity (8.1%)
**HSP-nMD** *(n* = *646)*	48.9%	17.1 ± 14.2 (range: < 1 mo – 70 yr)	33.3 ± 16.6 (range: < 1 mo – 82 yr)	79.6%	AR (57.1%) AD (30.1%) AR/AD (10.8%) X-linked (2.0%)	Gait disturbance (34.3%) Spasticity (28.1%) Learning/Cognitive problems (11.9%)
**Total (*N* = 1413)**	51.0%	18.7 ± 15.2 (range: < 1 mo–70 yr)	35.5 ± 17.4 (range: < 1 mo–90 yr)	79.3%	AR (57.7%) AD (19.8%) AR/AD (20.2%) X-linked (2.3%)	Gait disturbance (42.2%) Spasticity (18.1%) Learning/cognitive problems (9.9%)
p-value**	0.150	** < 0.001**	** < 0.001**	0.828	** < 0.001**	** < 0.001**

*HSP-MD*, hereditary spastic paraplegia (HSP) with movement disorder (MD); *HSP-nMD*, hereditary spastic paraplegia (HSP) without movement disorder (nMD); *AR*, autosomal recessive; *AD*, autosomal dominant; *yr*, year; *mo*, month

^*^Three most common initial presentations

^**^Statistical comparison between HSP-MD and HSP-nMD groups using chi-square with continuity correction or independent-samples *t* test

**Fig. 2:**
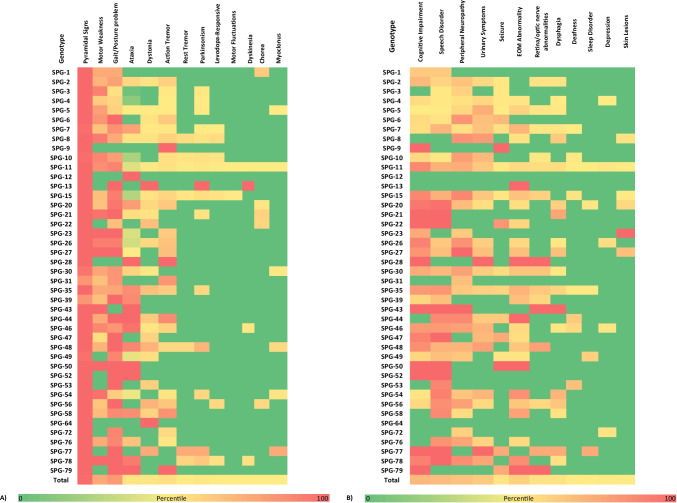
Heatmap of the prevalence of movement (**A**) and non-motor (**B**) disorders among different genotypes (SPGs) of hereditary spastic paraplegia (HSP) (cognitive impairment included dementia, mental retardation, learning disabilities; peripheral neuropathy included pes cavus, hammer toes, or NCS studies consistent with peripheral neuropathy; speech disorder included dysarthria, dysphasia) (EOM, extraocular movement)

A total of 43 different *SPG* genes were affected across the entire HSP group. In our dataset, *SPG11* (*n* = 391, 27.7%), *SPG7* (*n* = 256, 18.1%), *SPG4* (*n* = 171, 12.1%), and *SPG5* (*n* = 95, 6.7%) were the most frequently affected genes. Figure 2 provides a summary of the frequency of motor and non-motor features for each *SPG* subtype. While pyramidal signs were almost universally present, gait/posture problems were frequently reported in most genotypes except for *SPG9*, *SPG12*, *SPG28*, *SPG43*, and *SPG64*. Ataxia was highly prevalent in *SPG7*, *SPG12*, *SPG28*, *SPG43*, *SPG44*, *SPG46*, *SPG50*, *SPG52*, and *SPG79*, among others. Dystonia was reported very often in *SPG13* and *SPG64*. Regarding non-motor features, cognitive impairment and speech disorder were most commonly reported. Peripheral neuropathy and urinary symptoms were also frequently reported in various genotypes. Abnormalities in extraocular movements were commonly reported in *SPG13*, *SPG28*, *SPG44*, *SPG50*, and *SPG79*, while retinal and/or optic nerve abnormalities were frequent in *SPG28*, *SPG43*, and *SPG79*. Table 2 summarizes the commonest genotypes within each individual movement disorder. Ataxia was the most common movement disorder reported in 82.1% of recruited HSP population. SPG7 (36.8%), SPG11 (20.4%), and SPG5 (6.3%) were the commonest genotypes among those manifesting with ataxia. SPG11 was the most prevalent genotype in individuals presenting with action tremor (32.4%), dystonia (27.3%), parkinsonism (35.6%), rest tremor (52.9%), dyskinesia (66.7%), and myoclonus (40%). Majority of HSP cases manifested with chorea had SPG21 genotype (60%).

**Table 2:** Commonest SPGs genotypes in recruited individuals with hereditary spastic paraplegia (HSP) presenting with various movement disorders

Movement disorders	Frequency (%)	Commonest SPGs genotypes (%)
Ataxia	82.1%	SPG7 (36.8%) SPG11 (20.4%) SPG5 (6.3%)
Gait/postural problems	71.3%	SPG7 (32.4%) SPG11 (27.0%) SPG35 (3.5%)
Action tremor	18.4%	SPG11 (32.4%) SPG58 (9.9%) SPG7/SPG15/SPG26/SPG35 (7.2%)
Dystonia	9.1%	SPG11 (27.3%) SPG35 (20.0%) SPG7/SPG26 (7.3%)
Parkinsonism	7.5%	SPG11 (35.6%) SPG15 (11.1%) SPG35 (11.1%)
Rest tremor	2.8%	SPG11 (52.9%) SPG8/SPG10/SPG15/SPG78 (11.8%)
Dyskinesia	1.0%	SPG11 (66.7%)
Myoclonus	0.8%	SPG11 (40%)
Chorea	0.8%	SPG21 (60%)

Only SPGs with at least 2 cases are reported

Demographics and baseline characteristics of the HSP-MD cohort with various *SPG* genotypes are summarized in Supplementary Table 1. *SPG7* (31.2%) and *SPG11* (23.8%) were the most frequent genotypes in the HSP-MD group. Besides comprising the second most common genotype in the HSP-MD group, *SPG11* was the most frequent genotype identified in the HSP-nMD cohort (32.3%). Among other large genotype groups, *SPG4* (19.9% vs. 2.8%) and *SPG3* (4.7% vs. 0.5%) were more commonly associated with an HSP-nMD phenotype (Supplementary Fig. 1). As shown in Supplementary Fig. 2, among SPGs with at least 5 reported cases, *SPG78* presented only with an HSP-MD phenotype. Other genotypes that were more likely to present with MD were *SPG46* (95.2%), *SPG48* (85.7%), *SPG35* (84.8%), *SPG20* (84.2%), *SPG39* (83.3%), and *SPG58* (81.3%). Conversely, most cases with *SPG3* (92.3%), *SPG4* (89.5%), *SPG72* (85.7%), and *SPG47* (80.0%) presented with an HSP-nMD phenotype.

**Fig. 3:**
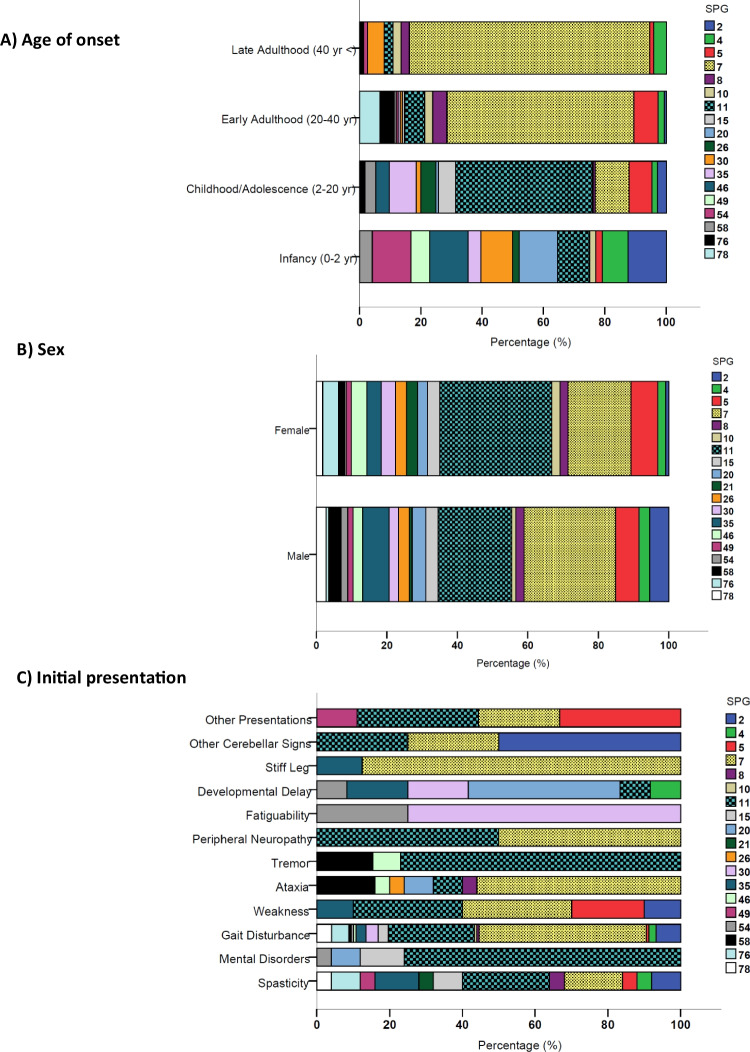
Frequency of different genotypes (SPGs) in patients with hereditary spastic paraplegia (HSP) manifested with a movement disorder (HSP-MD) and **A** various age of onsets, **B** different sexes, and **C** various initial presentation (*All SPGs have at least n* = *5 reported cases*)

In the HSP-MD cohort, patients with *SPG49* (0.3 ± 0.4 years) and *SPG20* (3.0 ± 5.5 years) genotypes had the youngest age of onset, while those with *SPG7* (34.4 ± 12.7 years) and *SPG8* (33.5 ± 14.2 years) were the oldest at the time of initial manifestation (Supplementary Table 1 and Supplementary Fig. 3). In this cohort, *SPG2*, *SPG20*, *SPG46*, and *SPG54* (11.8% for each) were the commonest genotypes among those manifesting during infancy, while *SPG11* (43.6%) and *SPG7* (66.1%) were the most common genotypes in individuals with onset during childhood/adolescence and adulthood, respectively (Fig. 3A). As illustrated in Fig. 3A, frequency of *SPG7* in HSP-MD phenotype remarkably increases by the age of onset; with no *SPG7* case manifested during infancy, reaching a frequency rate as high as 72.5% of cases with late-adulthood onset (age > 40 years). Regarding sex distribution of different genotypes in the HSP-MD group, *SPG11* was more common in females (31.8% vs. 20.9%), while *SPG7* was more frequently reported in males (26.0% vs. 17.9%) (*p* = 0.007, Fig. 3B).

**Fig. 4:**
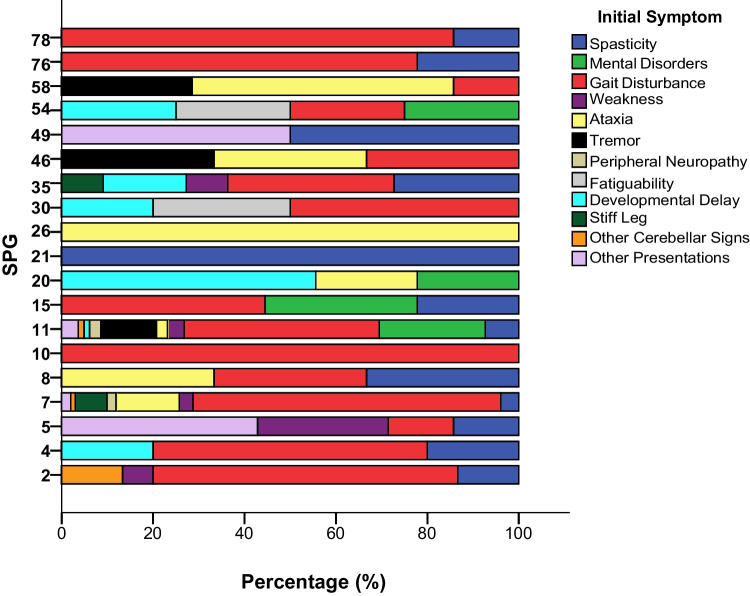
Frequency of various initial symptoms in patients with hereditary spastic paraplegia and a movement disorder (HSP-MD) with different genotypes (SPGs) (*All SPGs have at least n* = *5 reported cases*)

Notable variations in the initial presenting features according to different genotypes in the HSP-MD group were seen, as illustrated in Fig. 4. For instance, all patients with *SPG10* and *SPG21* presented with spasticity (100%) and gait disturbance (100%); all *SPG26* cases manifested initially with ataxia (100%). As expected, gait difficulty was commonly seen as the initial presenting symptom in several genotypes, especially in *SPG2*, *SPG4*, *SPG7*, *SPG11*, *SPG15*, *SPG30*, *SPG76*, and *SPG78* (> 50% prevalence). In most individuals with *SPG20*, developmental delay (55.6%) was the initial manifestation. The only genotypes in which tremor was reported as the initial manifestation were *SPG11*, *SPG46*, and *SPG58*. Figure 3C identifies the most frequent genotypes based on each initial presenting feature. In HSP-MD cases, SPG11 was the commonest genotype among those who initially manifested with tremor (76.9%) and learning/cognitive problems (76%). On the other hand, *SPG7* was the most common genotype reported in HSP-MD cases manifesting initially with stiff leg (87.5%), ataxia (56.0%), and gait disturbance (45.9%). Presentation at onset with cerebellar signs other than ataxia was more likely linked with *SPG2* (50.0%) (Fig. 3C).

**Fig. 5:**
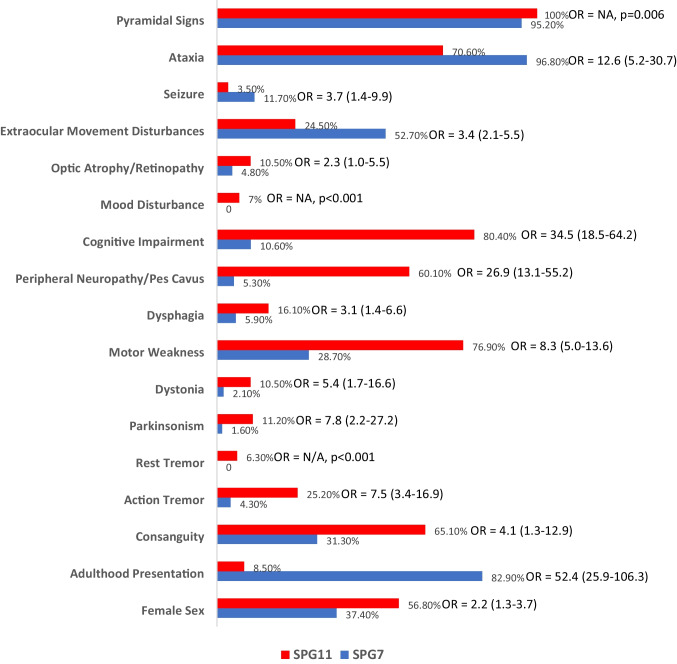
Significant differences in the prevalence of demographics and clinical features in patients with hereditary spastic paraplegia (HSP) manifested with a movement disorder (HSP-MD) with genotype SPG7 versus SPG11 (all corresponding odds’ ratios are calculated for the SPG with the higher prevalence rate) (OR, odds ratio; NA, not able to calculate OR due to 0 as denominator)

In addition to the age of onset, significant phenotypical differences were found between the two most common genotypes identified in the HSP-MD cohort, *SPG7* and *SPG11* (Fig. 5). Disease duration at the time of clinical assessment was longer in patients with *SPG7* (19.3 (SD = 13.9) years vs. 15.9 (SD = 9.0) years, *p* = 0.014). HSP-MD patients with *SPG7* had significantly higher likelihood of presenting with ataxia (96.8% vs. 70.6%, OR = 12.6 (5.2–30.7), adjusted OR = 11.7 (4.4–31.0), *p* < 0.001); extraocular movement disturbances (52.7% vs. 24.5%, OR = 3.4 (2.1–5.5), adjusted OR = 3.1 (1.9–5.1), *p* < 0.001); and seizure (11.7% vs. 3.5%, OR = 3.7 (1.3–9.9), adjusted OR = 4.2 (1.5–11.5), *p* = 0.005) compared to those with *SPG11*, whereas *SPG11* mutants showed significantly more frequent history of consanguinity in the family (65.1% vs. 31.3%, OR = 4.1 (1.3–12.9), *p* = 0.012), parkinsonism (11.2% vs. 1.6%, OR = 7.8 (2.2–27.2), adjusted OR = 5.7 (1.6–20.3), *p* = 0.007); dystonia (10.5% vs. 2.1%, OR = 5.4 (1.7–16.6), adjusted OR = 4.5 (1.4–14.3), *p* = 0.010); pes cavus/peripheral neuropathy (60.1% vs. 5.3%, OR = 26.9 (13.1–55.2), adjusted OR = 30.8 (14.0–67.7), *p* < 0.001); dysphagia (16.1% vs. 5.9%, OR = 3.1 (1.4–6.6), adjusted OR = 3.4 (1.5–7.8), *p* = 0.004); cognitive impairment (80.4% vs. 10.6%, OR = 34.5 (18.5–64.2), adjusted OR = 36.9 (18.6–73.3), *p* < 0.001); depression (7.0% vs. 0, *p* < 0.001); and retinopathy/optic nerve atrophy (10.5% vs. 4.8%, OR = 2.3 (1.0–5.5), adjusted OR = 2.5 (1.0–6.2), *p* = 0.050) compared to *SPG7*. All differences remained statistically significant after multivariate adjustment for disease duration (adjusted ORs).

**Fig. 6:**
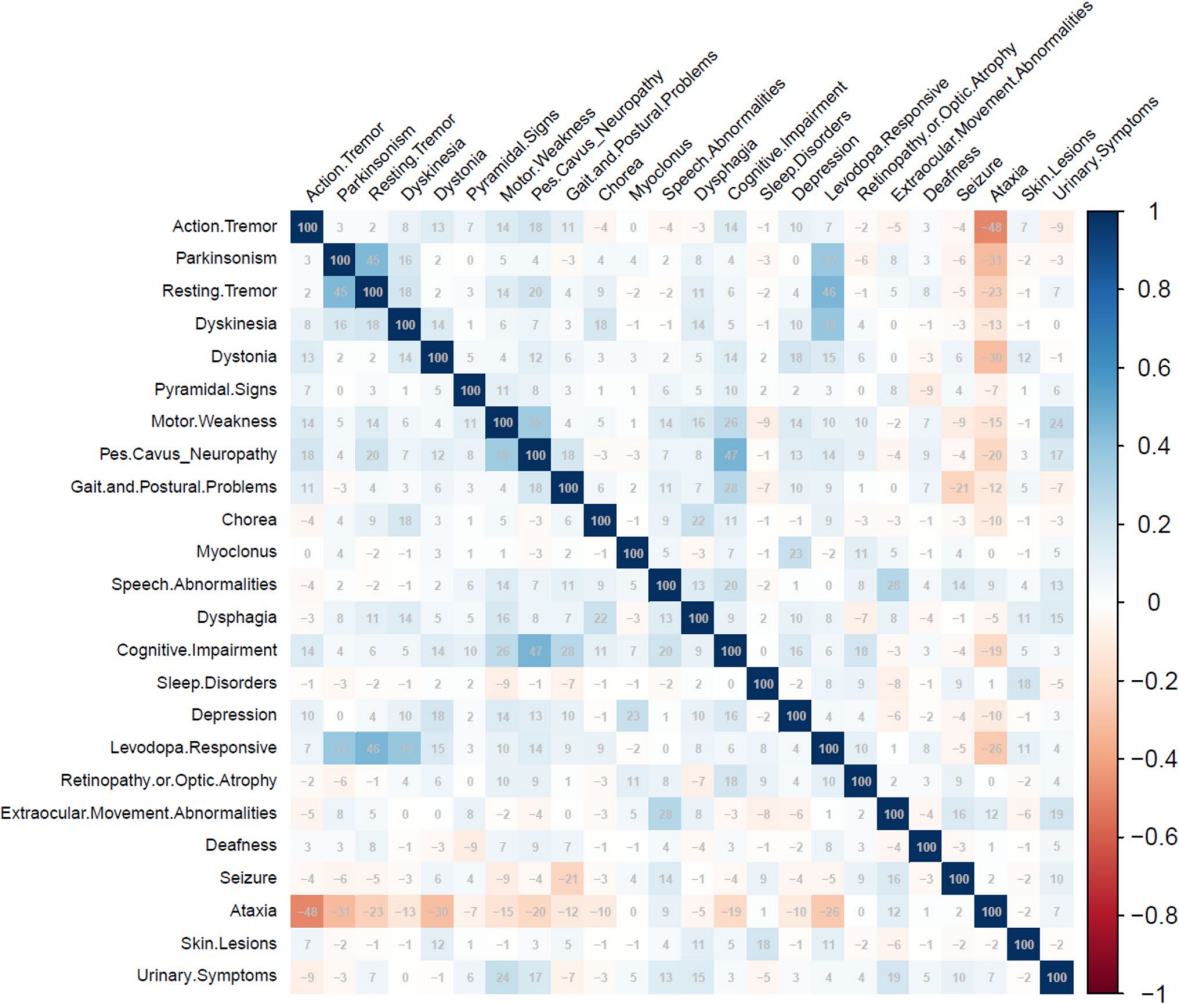
Correlation matrix of the associations between various presentations in patients with hereditary spastic paraplegia (HSP) manifested with a movement disorder (HSP-MD) (Corresponding Kendall’s correlation coefficient is presented as a percentage in each cell)

A correlation matrix of the inter-associations between various clinical features in patients with HSP-MD is shown in Fig. 6. In our exploratory analysis, cognitive impairment accompanies with higher prevalence of pes cavus/peripheral neuropathy (*r* = 0.47, *p* < 0.001). Among other significant correlations were direct association between dysphagia and chorea (*r* = 0.22, *p* < 0.001), myoclonus and depression (*r* = 0.23, *p* < 0.001), and extraocular movement abnormalities with urinary symptoms (*r* = 0.19, *p* < 0.001) and speech disorders (*r* = 0.28, *p* < 0.001). Ataxia inversely correlated with action tremor (r =  − 0.48, *p* < 0.001), parkinsonism (*r* =  − 0.31, *p* < 0.001), dystonia (r =  − 0.30, *p* < 0.001), resting tremor (*r* =  − 0.23, *p* < 0.001), pes cavus/peripheral neuropathy (*r* =  − 0.20, *p* < 0.001), and cognitive impairment (*r* =  − 0.19, *p* < 0.001).

## Discussion

HSP is a heterogeneous group of genetic disorders with a vast genotypic and phenotypic variability. Although there have been few studies on genotype–phenotype correlations in HSP [20, 21], details on the presence and type of MD in HSP are lacking. Furthermore, there is a dearth of knowledge on less frequent genotypes as these rarer variants are sparsely published as case reports and have been excluded from the only previously published conventional meta-analysis [20]. Implementing an IPD-level meta-analysis enabled us, for the first time, to compare demographics and clinical features between the widest variety of HSP genotypes manifesting with or without a movement disorder, to analyze genotype–phenotype associations in detail, and to provide important diagnostic clues even for the rarer variants by pooling data from case reports and case series.

In line with previous studies, we showed that HSP has a wide age at onset, ranging from a few months to 73 years [21]. Significant differences were seen according to genetic subtype, but variability may occur even within the same genotype. For instance, members of the same family with *SPG4* can present with SPG symptoms at very distinct decades in life [22]. In our study, the HSP-MD group had later age at onset and less frequent autosomal dominant inheritance than the HSP-nMD group. This could be in part related to the higher frequency of *SPG3* in the HSP-nMD group. A recent meta-analysis suggested that *ATL1* mutations (*SPG3*) are associated with earlier age at onset compared to other autosomal dominant forms such as SPAST (*SPG4*) and REEP1 (*SPG31*) [20]. The same finding has been shown in a previous study [23].

As may be expected, gait disturbance was the most frequent initial presentation. It is known that spasticity associated with HSP may only be evident on walking [22, 24]. Ataxia was the initial presentation in 8.7% of the HSP-MD group, and all *SPG26* cases manifested initially with ataxia (100%). Although ataxia is variably present during the course of *SPG26*, it has not been previously recognized as a major presenting feature in other studies analyzing the entire *SPG26* population (with or without MD). Ataxia was common at onset in *SPG7*, *SPG8*, *SPG20*, *SPG46*, and *SPG58*. The *SPG* genotype classically associated with ataxia in the literature is *SPG7* [5]. We found that early cognitive impairment and learning disabilities could be initial manifestations in HSP-nMD patients. *SPG4*, *SPG20*, *SPG30*, *SPG35*, and *SPG54* are the genotypes that can manifest initially with cognitive developmental delay. In our pooled IPD meta-analysis, early cognitive impairment was the most common initial presentation in *SPG20*, while in previous literature, SPG54 is known to more commonly present with cognitive abnormalities [20].

Our analysis also demonstrated several other genotypic differences between the two major phenotypic groups, with and without MD. *SPG11* was the most frequent genotype in the entire HSP group and in the HSP-nMD subgroup and the second most common in the HSP-MD group. The two most frequent genotypes in the HSP-MD group, *SPG7* and *SPG11*, were identified in nearly half of the cases with adulthood onset and childhood/adolescence onset, respectively, suggesting that age at onset is an important clue. Apart from age of onset, our analysis revealed numerous phenotypic features that help differentiating *SPG7* and *SPG11*. Individuals with SPG7 are more likely to develop ataxia, extraocular movement disturbances and seizure, whereas those with SPG11 more commonly experience parkinsonism, dystonia, peripheral neuropathy, dysphagia, retinopathy/optic nerve atrophy, cognitive, and mood impairment. Although some of our findings were previously shown in original cohorts (e.g., higher frequency of dysphagia and cognitive deficit in *SPG11* [21]), others are not necessarily in keeping with previous literature; for instance, frequency of parkinsonism in *SPG7* (21% in one study[25] versus < 2% in our report). A possible reason for this discrepancy is that the majority of previous studies reported their findings based on small case series with limited statistical power.

Our IPD-level meta-analysis also suggests numerous others important diagnostic clues in HSP-MD based on genotype–phenotype correlations depicted in the heatmaps as well as post hoc descriptive analysis based on initial presentation and age at onset. For example, all patients with *SPG10* and *SPG21* presented initially with spasticity and gait disturbance; developmental delay (55.6%) was the initial manifestation in most individuals with SPG20; the only genotypes in which tremor was reported as the initial presenting symptom were *SPG11*, *SPG46*, and *SPG58*, yet *SPG11* was the commonest genotype among those who initially manifested with tremor (76.9%) and mental disorders (76.0%). Furthermore, the most common genotype that manifested initially with stiff leg (87.5%), ataxia (56.0%), and gait disturbance (45.9%) was SPG7; and onset presentation with cerebellar signs other than ataxia was more likely linked with *SPG2* genotype (50.0%). Correlation matrix showed novel interesting phenotypic patterns. For instance, cognitive impairment and learning disabilities frequently accompany pes cavus/peripheral neuropathy, dysphagia correlates with chorea, myoclonus is common in patients with mood disturbances, and extraocular movement abnormalities co-occur with speech disorders.

To our knowledge, our study is the first meta-analysis of IPD in the context of HSP, while a conventional meta-analysis has been recently published [20]. IPD-level meta-analysis is a useful method for disease entities such as HSP where the majority of the published literature consists of small sample-size case series or case reports. Its main advantage is to boost statistical power by pooling individual-level data from all reported genotypes and therefore avoiding exclusion of rarer SPGs from genotype–phenotype analysis, which are otherwise usually eliminated from conventional meta-analysis given low statistical power in their original studies. As such, our study is the most inclusive and comprehensive genotype–phenotype analysis of the SPG subtypes. Other strengths of an IPD-level meta-analysis is the ability to account for missing information at the individual level, enabling various original statistical methods for comparison and correlation analyses, and obtaining results for specific subgroups of participants [26]. Many of these genotypic-phenotypic associations in our study were not previously reported given lack of available data in an original small-size case series. Furthermore, our analysis is the first to focus on two major phenotypes of HSP, with and without a movement disorder.

We acknowledge several limitations. First and most importantly, since our main objective was to focus on HSP patients with a movement disorder as at least one major phenotypic trait, our search strategy was designed to be systematically inclusive only of individuals with HSP-MD and not the entire HSP population. Nevertheless, data from the subset of HSP-nMD patients collectively reported in the articles included in our study were gathered, and this subset of patients was used as a comparison group, assuming these cases are representative of the entire HSP-nMD population. This search strategy has potentially resulted in an under-representation of *SPG4*, which is one of the commonest genotypes of HSP in patients with no prominent movement disorder [27]. Second, handling missing data is quite challenging in an IPD-level meta-analysis due to considerable inconsistencies in reporting symptoms and signs between original reports and the lack of a standardized checklist for data gathering. In our analysis, we considered any missing data on a specific symptom as “not reported or unknown” rather than absence of the symptom. Also, we did not provide specific mutation-level genotype–phenotype correlations. Lastly, due to the huge amount of work to extract individual-level data for every single case from each article, we were not able to update our database after the second iteration of literature searching in June 2018; therefore, original studies published after that date were not included.

In conclusion, our systematic IPD-level meta-analysis provides several clinically relevant phenotypic clues regarding demographic and neurologic features for various HSP genotypes. Many of these genotype–phenotype associations are novel and reported for the first time. Recognition of these genotype–phenotype correlates facilitate diagnosis in resource-limited settings where genetic diagnosis is not available or when genotyping is inconclusive. While symptoms are not evenly distributed among different HSPs, a direct multi-gene testing panel is required. Future studies are required to explore whether specific HSP clusters exist, based on these genotype–phenotype correlations, that correspond to known or unknown SPG loci with common pathogenesis.

## Supplementary Information

Below is the link to the electronic supplementary material.

Supplementary file1 - Search Strategies (DOCX 34 KB)

Supplementary file2 - Supplementary Figure 1 (DOCX 34 KB)

Supplementary file3 - Supplementary Figure 2 (DOCX 32 KB)

Supplementary file4 - Supplementary Figure 3 (DOCX 276 KB)

Supplementary file5 - Supplementary Table 1 (DOCX 22 KB)

## Data Availability

Data is accessible upon request.
