# Supplementary material for: Movement disorders in hereditary spastic paraplegia (HSP): a systematic review and individual participant data meta-analysis
Source: Neurol Sci. 2022 Nov 28;44(3):947–59. doi: 10.1007/s10072-022-06516-8 (PMC9925593; doi:10.1007/s10072-022-06516-8)
Supplement: Supplementary file 1 — Supplementary file1 - Search Strategies (DOCX 34 KB) [file 10072_2022_6516_MOESM1_ESM.docx]

Search Strategies

Searches run in:

- Ovid Medline
- Ovid Embase
- Web of Science Core Collection

On:

- August 4, 2015
- September 16, 2016
- June 13, 2018

# Searches run August 4, 2015

- **Ovid MEDLINE(R)**<1946 to July Week 4 2015>
- **Ovid Embase**<1974 to 2015 August 03>
- **Web of Science Core Collection** SCI-EXPANDED, SSCI, A&HCI, CPCI-S, CPCI-SSH, ESCI

**Ovid MEDLINE(R)**<1946 to July Week 4 2015>

|  | **Searches** | **Results** |
| --- | --- | --- |
| 1 | Spastic Paraplegia, Hereditary/ | 903 |
| 2 | (heredit* adj3 spas* adj3 parapleg*).mp,kw. | 1209 |
| 3 | (heredit* adj3 spas* adj3 parapar*).mp,kw. | 151 |
| 4 | (spas* adj3 heredit* adj3 parapleg*).mp,kw. | 1209 |
| 5 | (spas* adj3 heredit* adj3 parapar*).mp,kw. | 151 |
| 6 | Muscle Spasticity/ge | 369 |
| 7 | Paraplegia/ge | 364 |
| 8 | 6 or 7 | 638 |
| 9 | limit 8 to yr="1966 - 1988" | 240 |
| 10 | spastic parapleg*.mp,kw. | 1902 |
| 11 | spastic parapar*.mp,kw. | 2431 |
| 12 | (SPG1? or SPG2? or SPG3? or SPG4? or SPG5? or SPG6 or SPG7 or SPG8 or SPG9).mp,kw. | 714 |
| 13 | (famil* adj3 spas* adj3 parapleg*).mp,kw. | 220 |
| 14 | (famil* adj3 spas* adj3 parapar*).mp,kw. | 56 |
| 15 | (spas* adj3 famil* adj3 parapleg*).mp,kw. | 220 |
| 16 | (spas* adj3 famil* adj3 parapar*).mp,kw. | 56 |
| 17 | (struempell* adj2 disease*).mp,kw. | 6 |
| 18 | or/1-5,9-17 | 4423 |
| 19 | remove duplicates from 18 | 4287 |
| 20 | exp animals/ not (exp animals/ and exp humans/) | 4084521 |
| 21 | 19 not 20 | 4107 |

**Embase**<1974 to 2015 August 03>

|  | **Searches** | **Results** |
| --- | --- | --- |
| 1 | (heredit* adj3 spas* adj3 parapleg*).mp. | 1328 |
| 2 | (heredit* adj3 spas* adj3 parapar*).mp. | 243 |
| 3 | (spas* adj3 heredit* adj3 parapleg*).mp. | 1328 |
| 4 | (spas* adj3 heredit* adj3 parapar*).mp. | 243 |
| 5 | spastic parapleg*.mp. | 4707 |
| 6 | spastic parapar*.mp. | 3207 |
| 7 | (SPG1? or SPG2? or SPG3? or SPG4? or SPG5? or SPG6 or SPG7 or SPG8 or SPG9).mp. | 939 |
| 8 | (famil* adj3 spas* adj3 parapleg*).mp. | 268 |
| 9 | (famil* adj3 spas* adj3 parapar*).mp. | 67 |
| 10 | (spas* adj3 famil* adj3 parapleg*).mp. | 268 |
| 11 | (spas* adj3 famil* adj3 parapar*).mp. | 67 |
| 12 | (struempell* adj2 disease*).mp. | 3 |
| 13 | or/1-12 | 6479 |
| 14 | Limit 13 to embase | 5659 |
| 15 | Remove duplicates from 14 | 5621 |
| 16 | (exp animals/ or exp animal experimentation/ or nonhuman/) not ((exp animals/ or exp animal experimentation/ or nonhuman/) and exp human/) | 5724973 |
| 17 | 15 not 16 | 5250 |

**Web of Science Core Collection**

TS=((heredit* NEAR3 spast* NEAR3 parapleg*) OR (heredit* NEAR3 spast* NEAR3 parapar*) OR (famil* NEAR3 spast* NEAR3 parapleg*) OR (famil* NEAR3 spast* NEAR3 parapar*) OR (spast* NEAR3 heredit* NEAR3 parapleg*) OR (spast* NEAR3 famil* NEAR3 parapleg*) OR (spast* NEAR3 heredit* NEAR3 parapar*) OR (spast* NEAR3 famil* NEAR3 parapar*) OR spastic parapleg* OR spastic parapar* OR (struempell* NEAR2 disease*) OR SPG1? OR SPG2? OR SPG3? OR SPG4? OR SPG5? OR SPG6 OR SPG7 OR SPG8 OR SPG9)

Indexes=SCI-EXPANDED, SSCI, A&HCI, CPCI-S, CPCI-SSH, ESCI

Timespan=All years

# Update Searches run September 16, 2016

- **Ovid MEDLINE(R)** 1946 to September Week 1 2016
- **Ovid Embase**1974 to 2016 September 15
- **Web of Science Core Collection** SCI-EXPANDED, SSCI, A&HCI, CPCI-S, CPCI-SSH, ESCI, September 16, 2015

**Ovid MEDLINE(R)**1946 to September Week 1 2016

|  | **Searches** | **Results** |
| --- | --- | --- |
| 1 | Spastic Paraplegia, Hereditary/ | 1024 |
| 2 | (heredit* adj3 spas* adj3 parapleg*).mp,kw. | 1349 |
| 3 | (heredit* adj3 spas* adj3 parapar*).mp,kw. | 164 |
| 4 | (spas* adj3 heredit* adj3 parapleg*).mp,kw. | 1349 |
| 5 | (spas* adj3 heredit* adj3 parapar*).mp,kw. | 164 |
| 6 | Muscle Spasticity/ge | 392 |
| 7 | Paraplegia/ge | 379 |
| 8 | 6 or 7 | 673 |
| 9 | limit 8 to yr="1966 - 1988" | 239 |
| 10 | spastic parapleg*.mp,kw. | 2079 |
| 11 | spastic parapar*.mp,kw. | 2500 |
| 12 | (SPG1? or SPG2? or SPG3? or SPG4? or SPG5? or SPG6 or SPG7 or SPG8 or SPG9).mp,kw. | 782 |
| 13 | (famil* adj3 spas* adj3 parapleg*).mp,kw. | 226 |
| 14 | (famil* adj3 spas* adj3 parapar*).mp,kw. | 56 |
| 15 | (spas* adj3 famil* adj3 parapleg*).mp,kw. | 226 |
| 16 | (spas* adj3 famil* adj3 parapar*).mp,kw. | 56 |
| 17 | (struempell* adj2 disease*).mp,kw. | 7 |
| 18 | or/1-5,9-17 | 4670 |
| 19 | remove duplicates from 18 | 4557 |
| 20 | exp animals/ not (exp animals/ and exp humans/) | 4315088 |
| 21 | 19 not 20 | 4368 |

**Embase**1974 to 2016 September 15

|  | **Searches** | | | **Results** |
| --- | --- | --- | --- | --- |
| 1 | (heredit* adj3 spas* adj3 parapleg*).mp. | | | 1526 |
| 2 | (heredit* adj3 spas* adj3 parapar*).mp. | | | 265 |
| 3 | (spas* adj3 heredit* adj3 parapleg*).mp. | | | 1526 |
| 4 | (spas* adj3 heredit* adj3 parapar*).mp. | | | 265 |
| 5 | spastic parapleg*.mp. | | | 5183 |
| 6 | spastic parapar*.mp. | | | 3513 |
| 7 | (SPG1? or SPG2? or SPG3? or SPG4? or SPG5? or SPG6 or SPG7 or SPG8 or SPG9).mp. | | | 1079 |
| 8 | (famil* adj3 spas* adj3 parapleg*).mp. | | | 280 |
| 9 | (famil* adj3 spas* adj3 parapar*).mp. | | | 70 |
| 10 | (spas* adj3 famil* adj3 parapleg*).mp. | | | 280 |
| 11 | (spas* adj3 famil* adj3 parapar*).mp. | | | 70 |
| 12 | (struempell* adj2 disease*).mp. | | | 4 |
| 13 | or/1-12 | | | 7148 |
| 14 | (exp animals/ or exp animal experimentation/ or nonhuman/) not ((exp animals/ or exp animal experimentation/ or nonhuman/) and exp human/) | | | 5999807 |
| 15 | 13 not 14 | | | 6723 |
| 16 | limit 15 to embase | | | 5868 |
| 17 | remove duplicates from 16 | | | 5804 |
|  |  |  |  |  |

**Web of Science Core Collection**

TS=((heredit* NEAR3 spast* NEAR3 parapleg*) OR (heredit* NEAR3 spast* NEAR3 parapar*) OR (famil* NEAR3 spast* NEAR3 parapleg*) OR (famil* NEAR3 spast* NEAR3 parapar*) OR (spast* NEAR3 heredit* NEAR3 parapleg*) OR (spast* NEAR3 famil* NEAR3 parapleg*) OR (spast* NEAR3 heredit* NEAR3 parapar*) OR (spast* NEAR3 famil* NEAR3 parapar*) OR spastic parapleg* OR spastic parapar* OR (struempell* NEAR2 disease*) OR SPG1? OR SPG2? OR SPG3? OR SPG4? OR SPG5? OR SPG6 OR SPG7 OR SPG8 OR SPG9)

Indexes=SCI-EXPANDED, SSCI, A&HCI, CPCI-S, CPCI-SSH, ESCI

Timespan=All years

# Update searches run June 13, 2018 in:

- **Ovid MEDLINE(R) ALL**1946 to June 11, 2018
- **Ovid Embase**1974 to 2018 June 12
- **Web of Science Core Collection** SCI-EXPANDED, SSCI, A&HCI, CPCI-S, CPCI-SSH, ESCI, June 13, 2018

**Strategies**:

**Ovid MEDLINE(R) ALL**1946 to June 11, 2018

| **#** | **Searches** | **Results** |
| --- | --- | --- |
| 1 | Spastic Paraplegia, Hereditary/ | 1144 |
| 2 | (heredit* adj3 spas* adj3 parapleg*).mp,kw. | 1669 |
| 3 | (heredit* adj3 spas* adj3 parapar*).mp,kw. | 189 |
| 4 | (spas* adj3 heredit* adj3 parapleg*).mp,kw. | 1669 |
| 5 | (spas* adj3 heredit* adj3 parapar*).mp,kw. | 189 |
| 6 | Muscle Spasticity/ge | 416 |
| 7 | Paraplegia/ge | 389 |
| 8 | 6 or 7 | 711 |
| 9 | limit 8 to yr="1966 - 1988" | 237 |
| 10 | spastic parapleg*.mp,kw. | 2503 |
| 11 | spastic parapar*.mp,kw. | 2816 |
| 12 | (SPG1? or SPG2? or SPG3? or SPG4? or SPG5? or SPG6 or SPG7 or SPG8 or SPG9).mp,kw. | 963 |
| 13 | (famil* adj3 spas* adj3 parapleg*).mp,kw. | 237 |
| 14 | (famil* adj3 spas* adj3 parapar*).mp,kw. | 58 |
| 15 | (spas* adj3 famil* adj3 parapleg*).mp,kw. | 237 |
| 16 | (spas* adj3 famil* adj3 parapar*).mp,kw. | 58 |
| 17 | (struempell* adj2 disease*).mp,kw. | 7 |
| 18 | or/1-5,9-17 | 5396 |
| 19 | remove duplicates from 18 | 5372 |
| 20 | exp animals/ not (exp animals/ and exp humans/) | 4470062 |
| 21 | 19 not 20 | 5171 |
| 22 | limit 21 to yr="2016 -Current" | 622 |

**Embase**1974 to 2018 June 12 

| **#** | **Searches** | **Results** |
| --- | --- | --- |
| 1 | (heredit* adj3 spas* adj3 parapleg*).mp. | 1824 |
| 2 | (heredit* adj3 spas* adj3 parapar*).mp. | 305 |
| 3 | (spas* adj3 heredit* adj3 parapleg*).mp. | 1824 |
| 4 | (spas* adj3 heredit* adj3 parapar*).mp. | 305 |
| 5 | spastic parapleg*.mp. | 5839 |
| 6 | spastic parapar*.mp. | 3916 |
| 7 | (SPG1? or SPG2? or SPG3? or SPG4? or SPG5? or SPG6 or SPG7 or SPG8 or SPG9).mp. | 1316 |
| 8 | (famil* adj3 spas* adj3 parapleg*).mp. | 269 |
| 9 | (famil* adj3 spas* adj3 parapar*).mp. | 71 |
| 10 | (spas* adj3 famil* adj3 parapleg*).mp. | 269 |
| 11 | (spas* adj3 famil* adj3 parapar*).mp. | 71 |
| 12 | (struempell* adj2 disease*).mp. | 6 |
| 13 | or/1-12 | 8109 |
| 14 | (exp animals/ or exp animal experimentation/ or nonhuman/) not ((exp animals/ or exp animal experimentation/ or nonhuman/) and exp human/) | 6407221 |
| 15 | 13 not 14 | 7610 |
| 16 | limit 15 to yr="2016 -Current" | 1093 |

**Web of Science Core Collection**

TS=((heredit* NEAR3 spast* NEAR3 parapleg*) OR (heredit* NEAR3 spast* NEAR3 parapar*) OR (famil* NEAR3 spast* NEAR3 parapleg*) OR (famil* NEAR3 spast* NEAR3 parapar*) OR (spast* NEAR3 heredit* NEAR3 parapleg*) OR (spast* NEAR3 famil* NEAR3 parapleg*) OR (spast* NEAR3 heredit* NEAR3 parapar*) OR (spast* NEAR3 famil* NEAR3 parapar*) OR spastic parapleg* OR spastic parapar* OR (struempell* NEAR2 disease*) OR SPG1? OR SPG2? OR SPG3? OR SPG4? OR SPG5? OR SPG6 OR SPG7 OR SPG8 OR SPG9)

Timespan: 2016-2018.

Indexes: SCI-EXPANDED, SSCI, A&HCI, CPCI-S, CPCI-SSH, ESCI.
