# Supplementary material for: Movement disorders in hereditary spastic paraplegia (HSP): a systematic review and individual participant data meta-analysis
Source: Neurol Sci. 2022 Nov 28;44(3):947–59. doi: 10.1007/s10072-022-06516-8 (PMC9925593; doi:10.1007/s10072-022-06516-8)
Supplement: Supplementary file 2 — Supplementary file2 - Supplementary Figure 1 (DOCX 34 KB) [file 10072_2022_6516_MOESM2_ESM.docx]

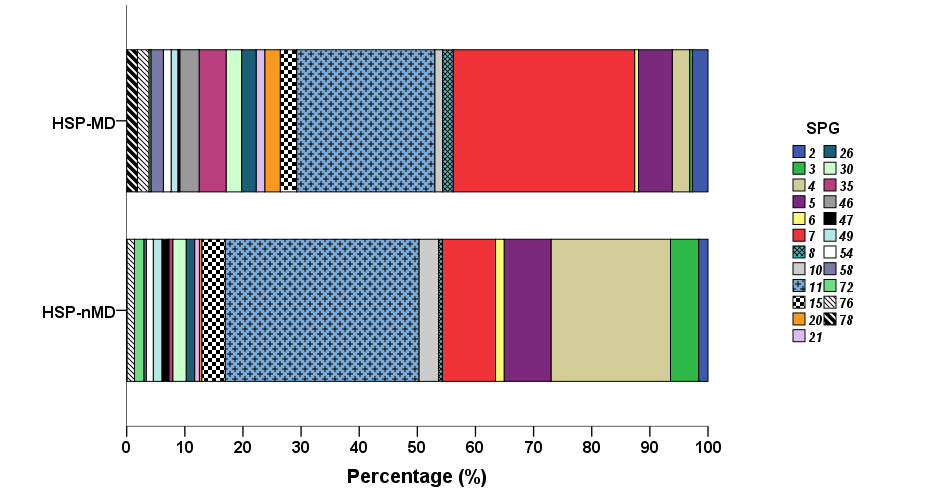


**SUPPLEMENTARY FIGURE 1.** Frequency of different genotypes (SPGs) of hereditary spastic paraplegia (HSP) in patients with (HSP-MD) and without (HSP-nMD) movement disorders (*All SPGs have at least n = 10 reported cases*)
