# Supplementary material for: Movement disorders in hereditary spastic paraplegia (HSP): a systematic review and individual participant data meta-analysis
Source: Neurol Sci. 2022 Nov 28;44(3):947–59. doi: 10.1007/s10072-022-06516-8 (PMC9925593; doi:10.1007/s10072-022-06516-8)
Supplement: Supplementary file 3 — Supplementary file3 - Supplementary Figure 2 (DOCX 32 KB) [file 10072_2022_6516_MOESM3_ESM.docx]

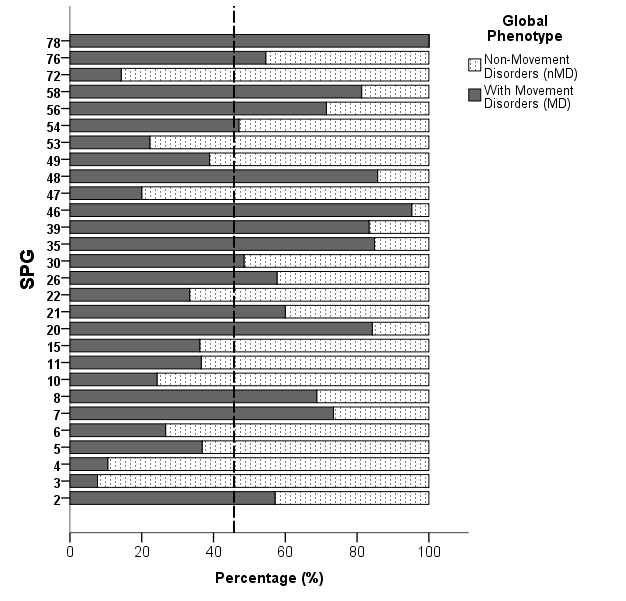


**SUPPLEMENTARY FIGURE 2.** Frequency of major global phenotypes (with or without a movement disorder) in patients with hereditary spastic paraplegia (HSP) with different genotypes (SPGs) (*All SPGs have at least n = 5 reported cases, dotted line demonstrates 45.7% as the overall prevalence of HSP-MD in the entire population*)
