# Supplementary material for: Movement disorders in hereditary spastic paraplegia (HSP): a systematic review and individual participant data meta-analysis
Source: Neurol Sci. 2022 Nov 28;44(3):947–59. doi: 10.1007/s10072-022-06516-8 (PMC9925593; doi:10.1007/s10072-022-06516-8)
Supplement: Supplementary file 4 — Supplementary file4 - Supplementary Figure 3 (DOCX 276 KB) [file 10072_2022_6516_MOESM4_ESM.docx]

Violin Plots of Age at Onset−year

All SPGs

SPG 78

SPG 76

SPG 58

SPG 54

SPG 49

SPG 46

SPG 35

SPG 30

SPG 26

SPG 20

SPG 15

SPG 11

SPG 10

SPG 8

SPG 7

SPG 5

SPG 4

SPG 2

0 10 20 30 40 50 60

Age at Onset (year)

**SUPPLEMENTARY FIGURE 3.** Violin plots for the distribution of the age of onset in different genotypes of hereditary spastic paraplegia (HSP) manifested with a movement disorder (HSP-MD)

(All SPGs have at least n = 5 reported cases)
