# Supplementary material for: Movement disorders in hereditary spastic paraplegia (HSP): a systematic review and individual participant data meta-analysis
Source: Neurol Sci. 2022 Nov 28;44(3):947–59. doi: 10.1007/s10072-022-06516-8 (PMC9925593; doi:10.1007/s10072-022-06516-8)
Supplement: Supplementary file 5 — Supplementary file5 - Supplementary Table 1 (DOCX 22 KB) [file 10072_2022_6516_MOESM5_ESM.docx]

| **SUPPLEMENTARY TABLE 1.** Demographic and baseline characteristics of different genotypes of hereditary spastic paraplegia (HSP) manifested with a movement disorder (HSP-MD) (*All SPGs have at least n = 5 reported cases*) | | | | | | | |
| --- | --- | --- | --- | --- | --- | --- | --- |
| **Genotype** | ***N*** *(%)* | **Sex**  Male *(%)* | **Age at Visit** (yr) *mean±SD* | **Age at Onset** (yr)  *mean±SD* | **Family History** *(%)* | **Consanguinity** *(%)* | **Initial Presentation***  *(%)* |
| *SPG-2* | 16 (2.7%) | 87.5% | 27.4±12.2 | 8.8±9.1 | 93.8% | 0 | Gait disturbance (66.7%)  Spasticity (13.3%)  Cerebellar signs (13.3%) |
| *SPG-4* | 18 (3.0%) | 61.5% | 37.2±21.1 | 20.1±21.9 | 81.8% | 0 | Gait disturbance (60.0%)  Spasticity (20.0%)  Developmental delay (20.0%) |
| *SPG-5* | 35 (5.8%) | 50.0% | 37.8±14.4 | 18.5±12.8 | 78.6% | 16.7% | Limb weakness (28.6%)  Falls (28.6%) |
| *SPG-7* | 188 (31.2%) | 62.6% | 52.9±12.4 | 34.4±12.7 | 70.0% | 31.3% | Gait disturbance (67.3%)  Ataxia (13.9%)  Limb stiffness (6.9%) |
| *SPG-8* | 11 (1.8%) | 54.5% | 53.7±10.7 | 33.5±14.2 | 100% | - | Spasticity (33.3%)  Gait disturbance (33.3%)  Ataxia (33.3%) |
| *SPG-10* | 8 (1.3%) | 37.5% | 51.2±22.2 | 26.5±16.4 | 100% | - | Gait disturbance (100%) |
| *SPG-11* | 143 (23.8%) | 43.2% | 28.7±9.0 | 13.0±7.4 | 76.4% | 65.1% | Gait disturbance (42.7%)  Learning/Cognitive problems (23.2%)  Tremor (12.2%) |
| *SPG-15* | 17 (2.8%) | 52.9% | 28.8±6.3 | 14.7±5.0 | 92.9% | 62.5% | Gait disturbance (44.4%)  Learning/Cognitive problems (33.3%)  Spasticity (22.2%) |
| *SPG-20* | 16 (2.7%) | 62.5% | 17.1±14.2 | 3.0±5.5 | 100% | 84.6% | Developmental delay (55.6%)  Ataxia (22.2%)  Learning/Cognitive problem (22.2%) |
| *SPG-21* | 9 (1.5%) | 22.2% | 48.0±9.9 | - | 88.9% | 100% | Spasticity (100%) |
| *SPG-26* | 15 (2.5%) | 53.3% | 43.9±14.2 | 6.6±2.9 | 84.6% | 60.0% | Ataxia (100%) |
| *SPG-30* | 16 (2.7%) | 43.8% | 35.7±27.4 | 21.9±24.5 | 57.1% | 50.0% | Gait disturbance (50.0%)  Fatiguability (30.0%)  Developmental delay (20.0%) |
| *SPG-35* | 28 (4.7%) | 67.9% | 18.4±14.1 | 6.4±7.6 | 65.4% | 46.2% | Gait disturbance (36.4%)  Spasticity (27.3%)  Developmental delay (18.2%) |
| *SPG-46* | 20 (3.3%) | 41.2% | 35.5±16.2 | 6.9±5.3 | 94.1% | 75.0% | Gait disturbance (33.3%)  Tremor (33.3%)  Ataxia (33.3%) |
| *SPG-49* | 7 (1.2%) | 57.1% | 11.8±6.0 | 0.3±0.4 | 83.3% | 28.6% | NR** |
| *SPG-54* | 8 (1.3%) | 83.3% | 30.5±23.8 | 11.1±18.9 | 75.0% | 37.5% | Gait disturbance (25.0%)  Fatiguability (25.0%)  Developmental delay (25.0%)  Learning/Cognitive problem (25.0%) |
| *SPG-58* | 13 (2.2%) | 69.2% | 30.2±13.1 | 10.5±8.4 | 100% | 70.0% | Ataxia (57.1%)  Tremor (28.6%)  Gait disturbance (14.3%) |
| *SPG-76* | 12 (2.0%) | 16.7% | 37.1±9.5 | 27.2±11.0 | 88.9% | 66.7% | Gait disturbance (77.8%)  Spasticity (22.2%) |
| *SPG-78* | 11 (1.8%) | 63.6% | 44.1±6.7 | 30.3±6.8 | 75.0% | 0 | Gait disturbance (85.7%)  Spasticity (14.3%) |
| **Total** | **591** | **53.6%** | **38.1±17.5** | **21.0±15.7** | **78.6%** | **57.1%** | **Gait disturbance (51.6%)**  **Spasticity (8.7%)**  **Learning/Cognitive problems (8.7%)**  **Ataxia (8.7%)** |

* Three most common initial presentations

** Not reported
